# Bergamo and Covid-19: How the Dark Can Turn to Light

**DOI:** 10.3389/fmed.2021.609440

**Published:** 2021-02-19

**Authors:** Norberto Perico, Stefano Fagiuoli, Fabiano Di Marco, Andrea Laghi, Roberto Cosentini, Marco Rizzi, Andrea Gianatti, Alessandro Rambaldi, Piero Ruggenenti, Carlo La Vecchia, Guido Bertolini, Stefano Paglia, Ferdinando Luca Lorini, Giuseppe Remuzzi

**Affiliations:** ^1^Istituto di Ricerche Farmacologiche Mario Negri, IRCCS, Bergamo, Italy; ^2^Gastroenterology Hepatology and Transplantation, Department of Medicine, ASST-Papa Giovanni XXIII, Bergamo, Italy; ^3^Dipartimento di Scienze della Salute, Università degli Studi di Milano, Unità di Pneumologia, Department of Medicine ASST-Papa Giovanni XXIII, Bergamo, Italy; ^4^Dipartimento di Scienze Medico Chirurgiche e Medicina Traslazionale, Sapienza Università di Roma, AOU Sant'Andrea, Rome, Italy; ^5^Emergency Department, ASST-Papa Giovanni XXIII, Bergamo, Italy; ^6^Infectious Diseases Department, ASST-Papa Giovanni XXIII, Bergamo, Italy; ^7^Pathology Unit, ASST-Papa Giovanni XXIII, Bergamo, Italy; ^8^Hematology Unit, ASST-Papa Giovanni XXIII, Bergamo, Italy; ^9^Department of Oncology and Oncohematology, Università degli Studi di Milano, Milan, Italy; ^10^Unit of Nephrology, Dialysis and Transplantation, Department of Medicine, ASST-Papa Giovanni XXIII, Bergamo, Italy; ^11^Department of Clinical Sciences and Community Health, Università degli Studi di Milano, Milan, Italy; ^12^Emergency Unit, Ospedale di Lodi, Lodi, Italy; ^13^Intensive Care Department, ASST-Papa Giovanni XXIII, Bergamo, Italy

**Keywords:** COVID-19 in Bergamo, Bergamo hospital challenges and reorganization, SARS-CoV-2 containment measures, lockdown and face masks, SARS-CoV-2 infection spread, Italian National Health Service, second wave of SARS-CoV-2 infection

## Abstract

The novel coronavirus, SARS-CoV-2, continues to spread rapidly. Here we discuss the dramatic situation created by COVID-19 in Italy, particularly in the province of Bergamo (the most severely affected in the first wave), as an example of how, in the face of an unprecedented tragedy, acting (albeit belatedly)—including imposing a very strict lockdown—can largely resolve the situation within approximately 2 months. The measures taken here ensured that Bergamo hospital, which was confronted with rapidly rising numbers of severely ill COVID-19 patients requiring hospitalization, was able to meet the initial challenges of the pandemic. We also report that local organization and, more important, the large natural immunity against SARS-CoV-2 of the Bergamo population developed during the first wave of the epidemic, can explain the limited number of new COVID-19 cases during the more recent second wave compared to the numbers in other areas of Lombardy. Furthermore, we highlight the importance of coordinating the easing of containment measures to avoid what is currently observed in other countries, especially in the United States, Latin American and India, where this approach has not been adopted, and a dramatic resurgence of COVID-19 cases and an increase in the number of hospitalisations and deaths have been reported.

## Introduction

The novel SARS-CoV-2 coronavirus continues to spread rapidly. On January 30, 2020 the WHO labeled it a public health emergency (1) and on July 21, 2020 the total number of laboratory-confirmed COVID-19 cases stood at over 14.7 million, having spread all over the world and caused over 611,000 deaths (2). The outbreak began in China and spread swiftly through Europe, the United States—where over 140,906 deaths had been reported as of July 21, 2020—and, more recently, Latin America (2).

**Figure 1:**
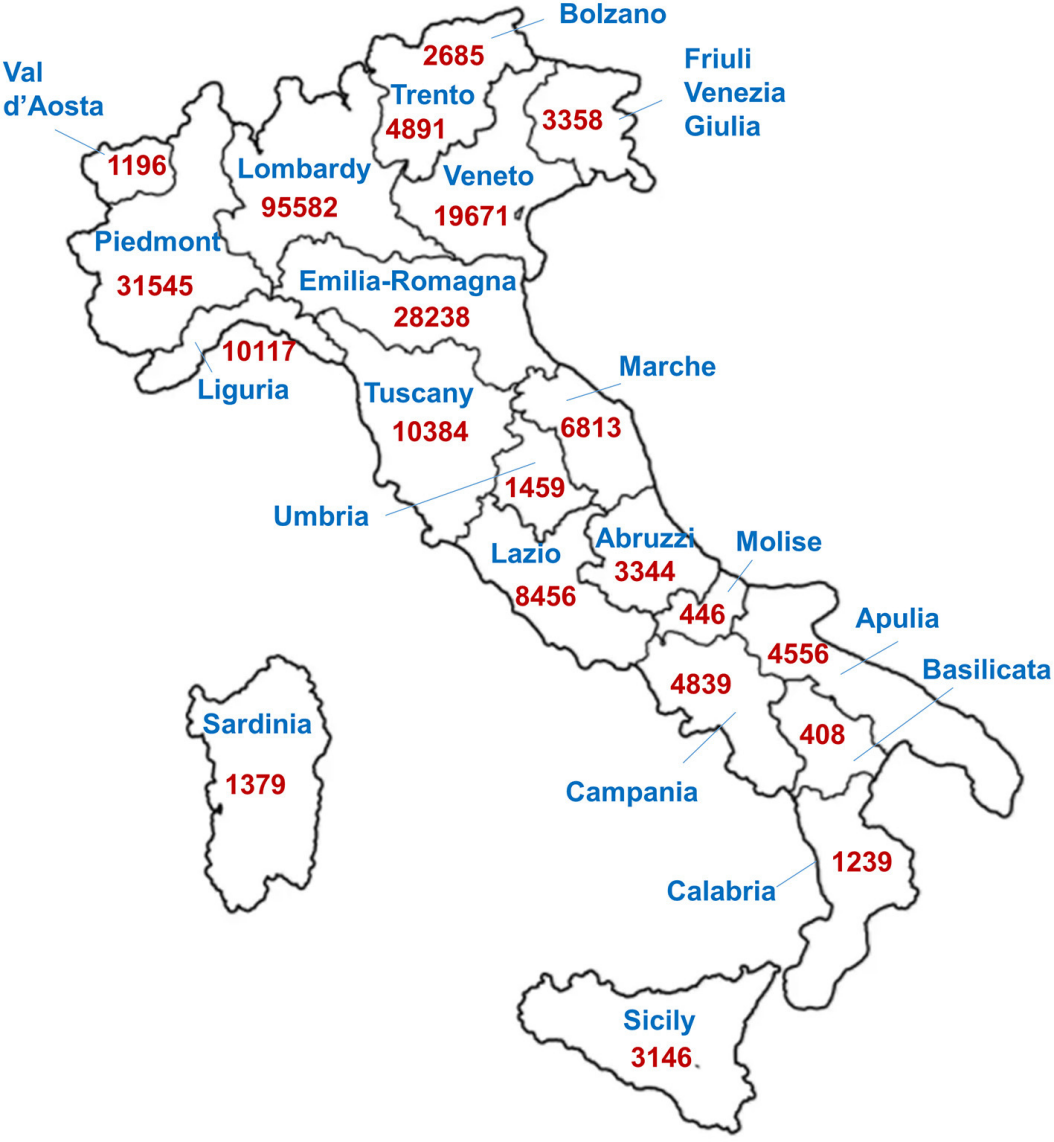
Reported laboratory-confirmed SARS-CoV-2 cases in the different regions of Italy (on 22 July 2020). (Data from Ministero della Salute, 22 July 2020).

Italy was among the first countries outside of Asia to report cases of COVID-19 (2) and by 21 July had reported 244,752 cases (35,023 deaths), with Lombardy the most severely affected region (95,582 cases, 16,797 deaths), particularly the province of Bergamo (14,865 cases) (Figure 1) (3). International responses to this emergency were neither immediate nor decisive, probably due to differing perceptions, which led to the seriousness of the situation being underestimated, resulting in responses with different levels of urgency. Furthermore, governments and healthcare systems in China, other Asian countries and Europe have different available resources.

In this article we describe (i) how initial differences between lockdowns in different areas of Lombardy and Bergamo province allowed SARS-CoV-2 to spread; (ii) what happened to a large tertiary hospital in Bergamo when measures to control the infection were not taken immediately across the territory; (iii) the key achievements 90 days later; and (iv) how local organization and awareness of the population helped to better contain COVID-19 in Bergamo province during the current second wave of the epidemic.

## Late Lockdown and Challenges to Hospitals

### Lombardy and Bergamo Vulnerable to the Spread of SARS-CoV-2

On January 30, 2020, the first COVID-19 cases in Italy were reported in two Chinese tourists. On February 21, 2020, the first case of COVID-19 in an Italian citizen was reported in Lombardy in a 38-year-old, otherwise healthy man living in the village of Codogno, in the province of Lodi, while he was visiting his relatives in nearby Castiglione d'Adda. It soon spread to nine other surrounding villages (together referred to as the Codogno cluster). SARS-CoV-2 was not initially suspected, and no special safety measures were adopted. The man had previously been in contact with colleagues in his multinational company in nearby Casalpusterlengo, participated in at least three meetings and dinners, ran in races outside of Lombardy, and met many people in his village before spreading the virus further at Codogno Hospital, where he was treated. This allowed the virus to circulate and spread. According to the patient's wife, he had been in contact with a friend who had recently returned from China (on January 21, 2020), who was initially considered the source of transmission. However, that person tested negative for SARS-CoV-2 and the index patient remains unknown.

In Lombardy, the genetic signature of the coronavirus was found to be very close to that called BavPat1 (differing by just one nucleotide in a genome comprising nearly 30,000 nucleotides) (4, 5). The BavPat1 viral genome had been found previously in a German man (6) admitted to the University Hospital in Munich on 24 January with symptoms of SARS-CoV-2 infection after being infected by an apparently healthy Chinese colleague who had traveled from China to Germany (7).

Since there were no travel restrictions in Europe at the time, and given the low level of concern regarding the first German case, the first person-to-person transmissions in Lombardy may be related to that German case. However, this remains controversial. Indeed, more recently, analysis of the viral genome data used to trace the transmission of the SARS-CoV-2 epidemic in Europe and the USA has challenged this view and suggested that the coronavirus was probably introduced to Italy (Lombardy) and the USA from China (8).

This would also mean that SARS-CoV-2 remained silent in Europe for several weeks, given that the Codogno cluster was identified approximately a month after the case in Munich. Two days after the first confirmed infection, the Italian government imposed social distancing rules and a local lockdown in the Codogno area. Two weeks later, the number of new infections began to decline.

Meanwhile, SARS-CoV-2 infection spread swiftly through other areas in Lombardy, particularly in the province of Bergamo, where the first cases were reported on February 23, 2020 in two small villages (Nembro and Alzano Lombardo) (Appendix Figure 1). Most patients were admitted to the Emergency Room at Alzano Hospital, a small provincial hospital. These individuals spread the infection to attending personnel and other patients and their relatives in the Emergency Room. The lower Seriana Valley, where Nembro and Alzano are located, is a highly industrialized area with business ties across Lombardy, other parts of Italy and abroad, including China, so a connection with the Codogno cluster was suspected once these patients were diagnosed. However, it later emerged that there may have been cases in the Seriana Valley even before the first case in Castiglione d'Adda was reported. The local press (and more recently *The New York Times*) (9) reported that a patient from Nembro was admitted to the Division of Medicine at Alzano Hospital on 15 February and initially not recognized as being infected with SARS-CoV-2. For 8 days, no special measures were taken to protect other patients, hospital personnel or visitors. The initial patient eventually tested positive for coronavirus on 23 February, 2 days before his death.

**Figure 2:**
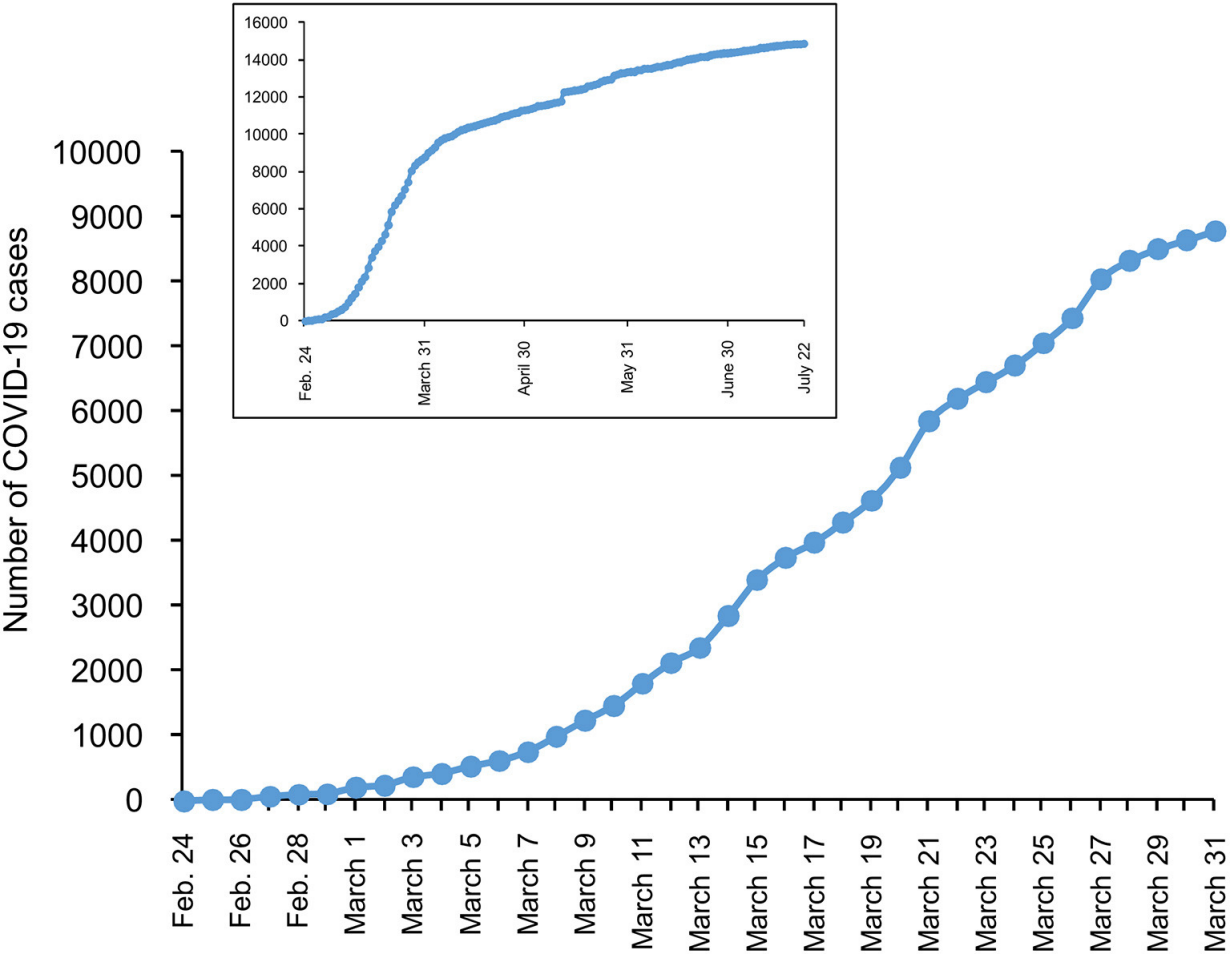
Number of laboratory-confirmed SARS-CoV-2 cases in Bergamo province from 24 February to 31 March 2020. Inset: number of SARS-CoV-2 cases up to 22 July 2020. (Data from Ministero della Salute, 22 July 2020).

Altogether, local circumstances, the large number of confirmed business ties with China and the daily movement of many workers through the Seriana Valley, as well as delays in activating adequate containment measures in Alzano Hospital, Nembro and Alzano, allowed the infection to spread swiftly to neighbor villages and the city of Bergamo (Figure 2). Unlike the Codogno cluster, Bergamo was not locked down promptly—following the cases reported on 23 February—but only on 9 March, 2 weeks later. By then, thousands of people had fallen ill, visited the Emergency Room and been admitted to Bergamo hospital, quickly pushing the hospital to its limits. This shows what can happen to a hospital—described below—when measures to contain an outbreak are not taken immediately across the territory. Italy was the first European country hit by the COVID-19 pandemic, and delays in deciding to implement a lockdown can be understood, if not justified. It is more difficult to justify the delay in taking such measures by most other major European countries (i.e., France, Spain, the UK), which were hit by the pandemic 2 or 3 weeks later and should have learned from the Italian experience. A Chinese modeling exercise in fact, suggested that COVID-19 could have been reduced by 86% if non-pharmacological interventions had been implemented 2 weeks earlier (10).

## Critical Reorganization of the Large Bergamo Hospital

The Bergamo hospital is a large tertiary hospital, a point of reference for high-need patients across Bergamo province (1,1 million inhabitants). Overall, it has 868 beds, 14 clinical departments including all specialties, six surgery departments, including a transplantation department, and intensive care units (ICU). Before COVID-19, in the period between February 1st and 20th, a total of 398 new patients were admitted to the hospital, with an average daily admission of 19.9 (mean admission over 5 consecutive days: 19). On February 23rd (baseline), 737 patients were hospitalized, 15 due to COVID-19. The rapidly rising numbers of COVID-19 patients forced the re-organization of Bergamo hospital, which is described below, from early interventions until the end of March, 2020. By 23 February, the General Director, Health Director, and the Heads of Departments had established a Crisis Unit that enabled the rapid reorganization and progressive channeling of infrastructural and human resources toward treating COVID-19. Dozens of patients were admitted daily, and 142 COVID-19 patients were admitted in one week. First the Infectious Disease Unit was completely rearranged to treat only COVID-19 patients, while other patients were moved or, where possible, discharged. Thereafter, separate COVID-19 units were created in both the adult and pediatric Internal Medicine and Surgery Departments, in ICUs and the semi-intensive area, and the Emergency Room. This reorganization occurred progressively in relation to the daily new COVID-19 admissions and it took 7 days to set up eight Units plus the SubAcute and ICU COVID-19 dedicated units. On 28 March, 498 of 779 beds were allocated to COVID-19 patients. Of these, 92 were admitted to the ICUs, and 12 to the sub-intensive critical area (11). Seventy percent of staff doctors were gradually redistributed to coronavirus units, regardless of specialty. On March 30th, over 400 physicians, 900 nurses and technicians were dedicated exclusively to the COVID-19 units. At this point, the nurse-to-patient (NPT) ratio was 1:1.5 in ICUs and the sub-intensive critical area (946 min per patient day), as well as 1:4 (vs. 1:7 in the pre-COVID-19 era) (361 min per patient day) in general COVID-19 wards. On average, throughout the month of March (day 1 to 31) the NPT ratio was 1:25 for ICUs and the sub-intensive critical area (1.178 min per patient day). One hundred and twelve new physicians and 102 nurses and technicians were hired over the course of the emergency. In parallel, several training meetings (small groups working face-to-face, on-line lessons and a dedicated video tutorial hosted on the hospital's intranet) were held every day that covered the use and need for personal protective equipment (PPE) and how to approach and manage COVID-19 patients, with a special focus on the management of respiratory insufficiency and the use of non-invasive ventilation support (12). These peer educational courses were provided to all personnel in the hospital, with over 1,500 people trained in 1 week. Oxygen consumption in the hospital increased from 140 m^3^/h to 680 m^3^/h, enabled by a technical adjustment to the oxygen system made within 3 days.

**Figure 3:**
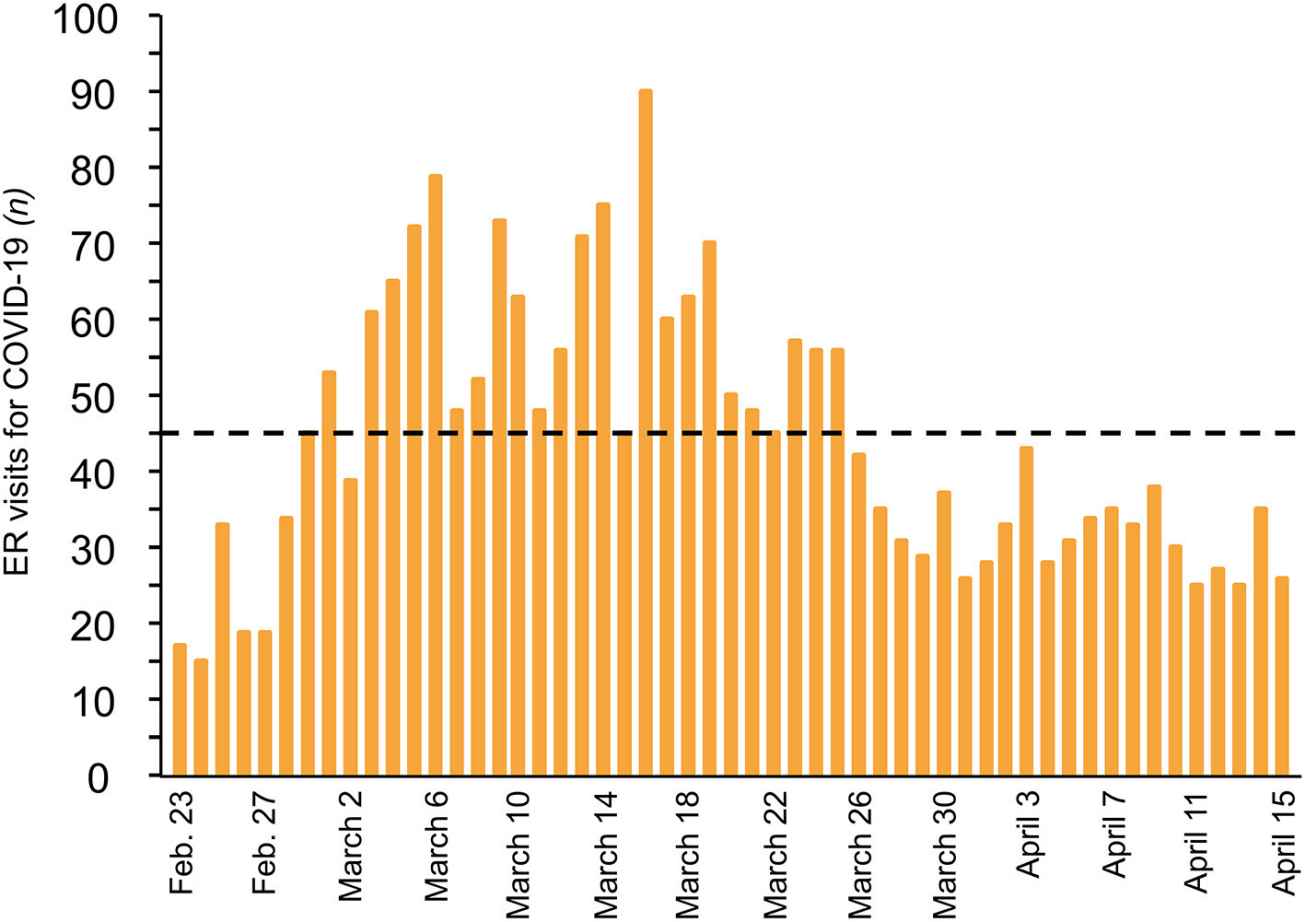
Daily visits for COVID-19 at Emergency Room of Bergamo hospital. (From 23 February to 15 April 2020). Dashed line indicates threshold of more than 45 daily visits.

The coordination of all COVID-19 emergency-related activities, from the Emergency Room (ER) to the ICU, was critical. In the ER, positive/suspected patients were separated from other patients, which was crucial for patients classified as emergency level at triage. In the COVID-19 positive/suspected area, early discharge pathways were soon activated to limit admission to those in urgent need of medium to high levels of care. An area in the ER was designated for immediate Continuous Positive Airway Pressure (CPAP)/Non-invasive Ventilation for a large number of patients while they awaited transfer to high-dependency units, and a Shock Room was established for intubated patients awaiting ICU beds. The number of operative physicians in the Emergency Department was increased from 33 to 45. A pre-triage station with very brief patient processing times was also established, where nurses with flow charts classified patients and advised non-urgent patients to return home (Appendix Figure 2). During the first step of triage, patients were divided into two different pathways (SARS-CoV-2 vs. “Clean non-SARS-CoV-2”) according to a check-list that included history, signs and symptoms. Initially, the SARS-CoV-2 area covered 20% of the ER and by the first week of March, the COVID-19 area had increased to 90%. Nurses also performed Quick Walking tests, which proved very sensitive in identifying initial pulmonary dysfunction in patients affected by COVID-19 (Appendix Figure 3). For 25 days, there were over 45 daily COVID-19 admissions to the ER, with a peak of 90 (Figure 3). In the ER, initial treatment focused on managing respiratory failure to reduce the likelihood of deterioration and the need for intubation. Even well-trained and experienced infectious disease specialists found themselves at a loss during the most overwhelming days of the outbreak. In the weeks following the first COVID-19 admissions, infectious disease specialists, while running their wards, also had to work closely with the Emergency Room to streamline patient intake as much as possible. However, the growing number of COVID-19 patients also made it necessary to distribute infectious disease specialists to different wards to support the growing number of teams comprising doctors with different expertise and specializations.

Many tasks were also managed by equally overworked pneumologists. Their priority was to manage the sudden arrival of large numbers of patients, which overwhelmed the capacity of the Respiratory Unit. The pneumologists supported the new COVID teams and, alongside other specialists, developed flow charts. These were sometimes arbitrary, due to the inevitable lack of evidence. Pneumologists were crucial in setting up dedicated meetings for collegiate decision-making regarding patient management, which cannot be left to individual healthcare professionals as it may lead to burnout.

Radiologists, too, faced a completely unexpected emergency in the form of the interstitial pneumonia caused by this new virus. At admission, radiologists were called to detect and try to characterize and stage the disease for each patient; and once the diagnosis was established, they were asked to assess disease progression or resolution. Radiologists encountered both diagnostic and organization challenges. At onset, the disease presents with peripheral, posterior “ground-glass” opacities in the lower lung lobes, which are commonly overlooked in chest X-rays (13). High-resolution Computed Tomography (CT), although extremely useful because of its high sensitivity, shows typical but not pathognomonic findings, further complicating the diagnostic process (14).

During the first 2 weeks, the exponential growth of SARS-CoV-2-related acute respiratory failure forced the hospital to re-organize ICUs so more beds could become COVID-ICU beds (11). A team of mixed intensive care physicians, from neuro-, cardio- and general adult ICUs, was created to manage the unfolding nightmare. They also monitored and constantly re-evaluated patients with mild to moderate respiratory failure in other COVID-19 units, to determine the best time for instituting CPAP helmets or non-invasive ventilation and, in the worst cases, transfers to the ICU for intensive care. By March 9, 49 ICU patients needed mechanical ventilatory support (11). The percentage of intubated patients increased progressively until all ICU beds were occupied by intubated and mechanically ventilated patients (11). When no more ICU beds were available, patients were transferred to other Italian and European hospitals. Almost all elective surgeries were canceled within the first week, and only 2 of the 28 operating theaters remained open for urgent general and cardiac surgeries.

Meanwhile, outpatient services continued for patients with priority access, for dialysis, oncology visits and services, and non-deferrable obstetric assistance (between February 22 and March 30, over 422 children were born at the hospital). As far as risk containment procedures, separate paths for SARS-CoV-2 positive and negative patients were created. Moreover, for all patients who needed hospital admission (either for planned admissions or emergency admissions) throat swab molecular testing was routinely performed within a maximum of 72 h. For all outpatients, a check point to collect medical history and epidemiological risk was created.

## Key Achievements

**Figure 4:**
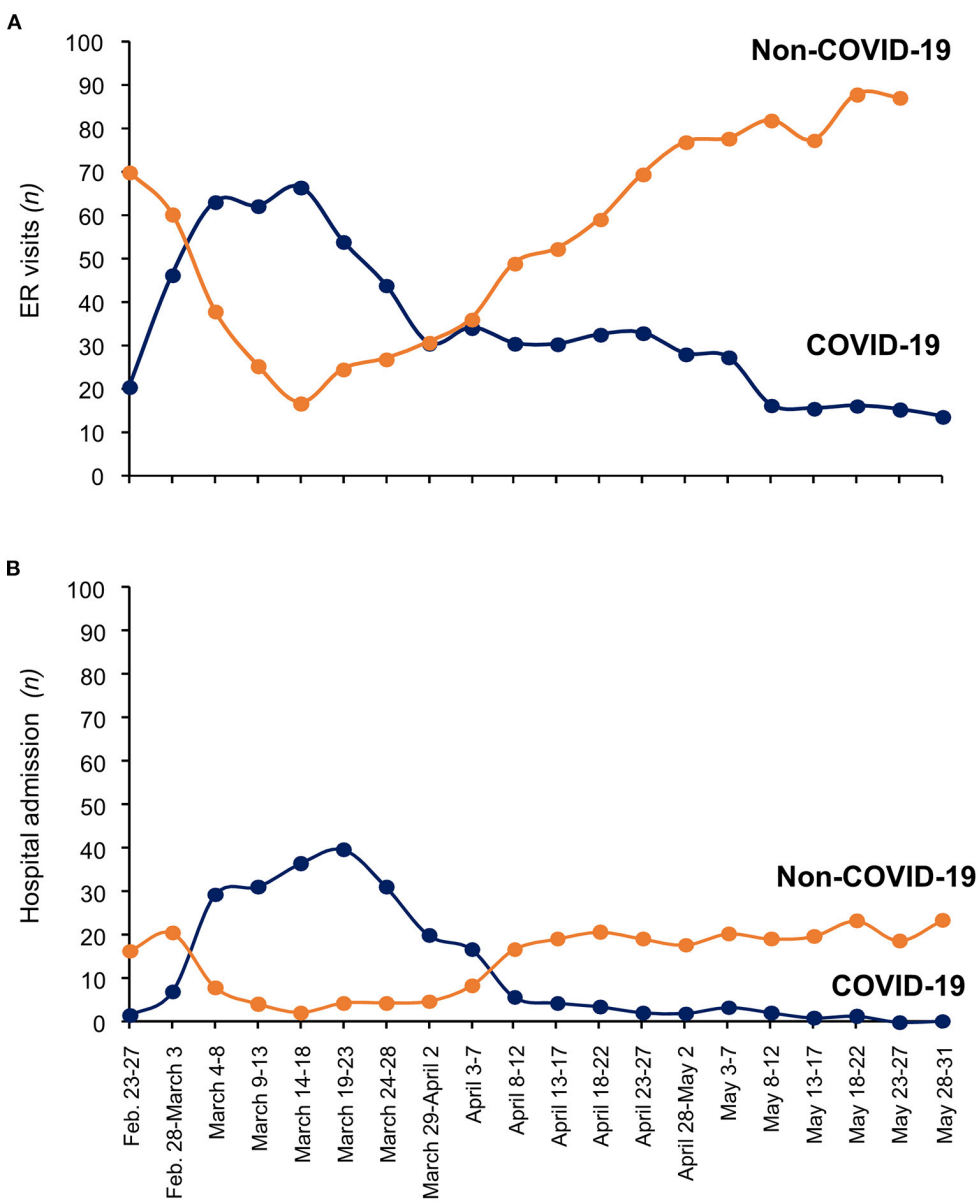
Emergency Room visits **(A)** and Hospital admission **(B)** at Bergamo hospital (from 23 February to 31 May 2020). Each point represents mean visits/admissions during 5 consecutive days. Blue line and orange line indicate COVID-19 and non-COVID-19 visit/admission, respectively.

The reorganization of the hospital made it possible to cope with the unprecedented drama that unfolded toward the end of March. This is highlighted by the number of visits to the ER and hospital admissions between February and May (Figures 4A,B). During this time, the daily overall number of ER visits remained relatively stable, on average 91 per day (95% CI 87.4–94–6). However, a few days after February 23rd, daily ER visits due to COVID-19 increased rapidly from 17 to 53 (March 1st), while non-COVID-19 visits declined from 79 to 49 over the same period (Figure 4A). Thereafter, daily COVID-19 ER visits continued to increase, with a peak of 90 on March 16th. By the end of March this trend had stopped, and COVID-19 ER visits started to decline, averaging 31.9 (±1.1) a day in April, and 18.1 (±1.2) a day in May. A similar profile was found for daily hospital admissions due to COVID-19 (Figure 4B), with a peak of 50 patients on March 17th. Notably, until the end of March, daily hospital admissions after triage equaled, on average, half of the daily COVID-19 ER visits (48% ± 4.7 SE), but then declined to 22.1% (±3.7) and 8.5% (±1.9), in April and May, respectively. From May 23 no more COVID-19 patients were admitted, despite an average 14.7 (±0.8) daily COVID-19 ER visits until May 31st (Figures 4A,B). This may reflect progressive improvements in disease severity after March, so patients who visited the ER were less sick, did not require admission and could be managed at home. Overall, between February 23rd and May 31st, 1,944 COVID-19 patients were admitted to the hospital, 1,376 of them by the end of March. Notably, by July 10th, no further COVID-19 patients with classical manifestations of the disease had been admitted to Bergamo hospital, and only two COVID-19 patients, who were slowly recovering, remained in the ICU. In parallel, Bergamo province (15), especially the small community of Nembro (16), experienced an unprecedented number of deaths compared to numbers for the same period (February-April) in previous years (2015–2019), which returned to normal levels in May. Similar findings and a similar profile of death burden were reported at the national level across Italy (17, 18).

## Containment of COVID-19 in Bergamo During the Current Second Wave of the Epidemic

**Figure 5:**
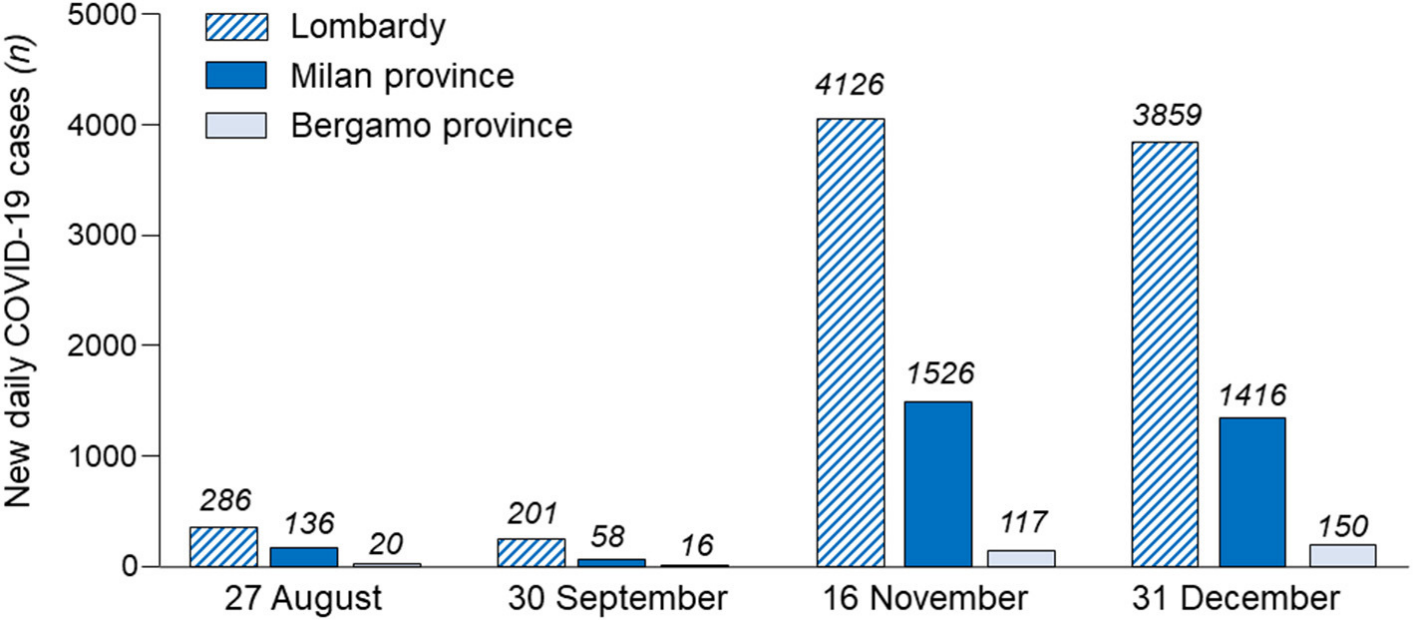
New daily cases in Lombardy, Milan province and Bergamo province during the period 27 August to 31 December 2020 (second wave of SARS-CoV-2 infection in Italy).

After the summer holidays, Italy exhibited a slight but progressive increase in the number of new daily positive COVID-19 cases from 1,119 (27th August) to 1,761 (30th September) (2, 19). From early October 2020, these numbers increased sharply, reaching a peak of 35,073 new daily cases on 16th November. With the reintroduction of containment measures, total daily cases in Italy declined to 13,978 by 31st December, and stabilized between 14,000 and 17,000 cases in early 2021. A similar profile of new daily COVID-19 cases has been shown for the Lombardy region (27th August: 286 cases; 30th September: 201 cases: 16th November: 4,128 cases; 31st December: 3,859). However, these averages obscure important local variations. Indeed, as shown in Figure 5, at the peak of the second COVID-19 wave in Lombardy (16th November), Bergamo province accounted for only 2.8% of new daily cases in the region, whereas the province of Milan accounted for 36.9%. Notably, between 1st September and 25th October 2020, cities/villages in Lombardy with excess deaths of least 0.5% (including those in Bergamo provinces) during the first COVID-19 wave (1st March to 30th June 2020), counted 216 cases per 100,000 people, 33% lower (654 cases per 100,000 people) than in areas with excess deaths of 0.1 to 0.2% of the population. As a result, during the second COVID-19 wave, Bergamo hospital easily handled the cases of moderate/severe COVID-19 that reached the ER or were then admitted to hospital units, including the ICU. Notably, during the second wave, hospitals in Bergamo were taking in patients from other parts of Lombardy.

## Discussion

There are three hypotheses, which are not mutually exclusive, to explain the improvements in clinical features of COVID-19 patients who visited the ER of Bergamo hospital, thus limiting and eventually eliminating the need for hospitalization by the end of May. The most immediate is the lockdown imposed in Lombardy, including in the Bergamo area, after March 8th, and the activation of viral infection containment measures, such as home isolation for symptomatic individuals and their relatives, physical distancing and the use of face masks for apparently healthy asymptomatic subjects when they began to move around their own locked-down areas for specific reasons, or when they were subsequently allowed to circulate more freely in the community after restrictions began to be eased (May 4th, 2020). This hypothesis is supported by findings from a recent systematic review and meta-analysis of 44 relevant, comparative non-randomized studies involving patients with COVID-19 or affected by the other betacoronaviruses that cause SARS and MERS (20). The data showed that there was a strong association between physical distancing of at least 1 meter and the optimum use of 12–16-layer cotton or surgical masks in the community and protection against person-to-person transmission of coronaviruses. This is in line with the recommendations of the Association of Schools of Public Health in the European Region, launched on 24th April, for the use of face masks to mitigate the risk of spreading SARS-CoV-2 in the community (21). Moreover, face masks have been found to significantly reduce influenza and coronavirus RNA in respiratory droplets and aerosol from children and adults with acute respiratory illness (22). Thus, these measures reduced the viral load transmitted person-to person, and possibly limited the localization of SARS-CoV-2 to the upper respiratory tract, resulting in mild or moderate symptoms. The second possibility is that SARS-CoV-2 has become milder over time, causing mainly a mild upper respiratory tract infection, as previously found with other common coronaviruses (23). However, doubts raised by some investigators about this hypothesis, and recent reports of second waves of SARS-CoV-2 infections emerging in some states in the US, as well as in Beijing (China), lessen our enthusiasm for this explanation for milder cases of COVID-19 being observed over time. In this context, the moderate increase in the mean temperature (between February 2020, 7.7°C, and May 2020, 18.1°C) and the relatively stable absolute humidity (February 2020: 61.3%, and May 2020: 65.7%) in the Bergamo area rule out the possibility that local climate condition changes contributed to attenuating the clinical presentation of COVID-19. Incidentally, although the issue of the seasonality of SARS-CoV-2 is debated (24–26), a prospective cohort study of 144 geographical areas worldwide showed that temperature and absolute humidity were not associated with the epidemic spread of COVID-19. Instead, the number of public health interventions (i.e., restriction of mass gatherings, school closures and social distancing measures) was inversely associated with epidemic growth (25).

The third possibility is that the very high rate of infection during the first wave in Bergamo province has conferred a measure of immunity. This is supported by the findings of a cross-sectional study we performed to assess the prevalence of SARS-CoV-2 infection, involving 423 workers in Bergamo province who returned to the workplace after the end of the Italian lockdown on June 3, 2020, (27). Among them cohort, the seroprevalence of SARS-CoV-2 infection was 38.5% (163 positive subjects over 423 tested), significantly higher than that reported in other badly hit areas in the world, including New York (19.8%) and London (17.5%) (28). Notably, seroprevalence was even higher in subjects living in Nembro—one of the villages in Bergamo province where the infection began to spread—with a 56.7% positivity rate, compared to Bergamo city (37.7%) and other areas of the province (36.9%). The longevity of the humoral response to SARS-CoV-2 is heterogenous. In some cases, it has been reported that circulating antibody titers declined 4 months after symptom onset, especially in mild cases (29–31); others exhibited relatively stable anti-SARS-CoV-2 antibodies at 4 months (32) or over more than 6 months (33) after onset of initial COVID-19 symptoms. In the cross-sectional study involving the population of Bergamo province (27), seroprevalence was assayed to 3 months after symptom onset, conceivably indicating that the 38.5% seroprevalence rate did reflect the minimum prevalence of infection in the area. However, the cumulative prevalence could have been even higher, considering that a large subset of subjects may have memory B cells, CD4^+^ T cells and CD8^+^ T cell memory against SARS-CoV-2, without detectable levels of circulating anti-SARS-CoV-2 antibodies (33–35). This would suggest that Bergamo province was moving toward natural a degree of herd immunity after the first wave of the epidemic, which would explain the limited number of new COVID-19 cases in the more recent second wave, compared to numbers in other areas of Lombardy.

In conclusion, as early as January 2020, the WHO shared detailed information about a cluster of cases of pneumonia of unknown cause with all member states and advised them to take precautions to reduce the risk of this acute respiratory infection spreading (36). However, Western countries ignored the warnings about the COVID-19 pandemic and wasted over a month before taking action to curb the spread of the virus. Meanwhile, in Italy the protocol from the Ministry of Health suggested that nasopharyngeal swabs for SARS-CoV-2 be performed only in individuals arriving from regions in China affected by COVID-19. Consequently, in late February, Italy—in particular Lombardy and the Bergamo area, which experienced the first major outbreak of SARS-CoV-2 infection—saw the number of COVID-19 cases rise rapidly, quickly overwhelming hospitals. Nevertheless, the lesson from Italy is that, despite not being prepared for this unprecedented drama, the necessary action to deal with the coronavirus was taken (albeit belatedly), and the outbreak peaked a few weeks later but then declined rapidly. The institution and maintenance of a very strict lockdown, coupled with the mandatory use of face masks, the prohibition of mass gatherings, school closures and the implementation of social distancing measures, all contributed to containing the epidemic and largely solved the problem within 7 weeks, as observed in Bergamo (37). This was also achieved thanks to appropriate coordination in applying stringent containment measures across Italy. Notably, Italy maintained the lockdown measures until the daily number of laboratory-confirmed new COVID-19 cases was relatively low and was then cautious about reopening. Thus, after a week of relative stabilization or even a decline in daily COVID-19 cases in Italy, on 4th May manufacturing and sales activities restarted, with some limitations. Only on 3rd June were bars/restaurants, cinemas, concert venues, parks and playgrounds for children allowed to reopen, with limitations regarding the number of participants/visitors permitted. Nonetheless, since infectious diseases ignore provincial, regional, and national borders, it is essential to coordinate the relaxation of containment measures to limit community transmission and a resurgence of COVID-19, as shown in Italy since October, and supported by findings from a meta-population model of COVID-19 transmission in Europe (38). The importance of communities coordinating the easing of various containment measures for COVID-19 is highlighted by what can currently be observed in the Unites States, where this approach has not been adopted and a dramatic resurgence of COVID-19 cases and an increase in the number of deaths have been reported across the country (2). Furthermore, we should not forget the role played in Italy by the Italian National Health Service (SSN), established in the late 1970s (39), which extended the right of universal access to uniform healthcare services to all citizens and foreign, legal residents, based on the principle of universal coverage, solidarity and equity. During the COVID-19 outbreak this contributed to the provision of high-quality care for—importantly—all patients in need, despite the difficulties created by the unprecedented outbreak. For the future, the Bergamo experience has taught us i) how to rapidly reorganize the main hospitals for any potential future outbreaks, and ii) the need, across the territory, to better support and interact with primary care physicians in the community as well as with doctors, nurses and other healthcare personnel operating in nursing homes, by providing them with simple and clear recommendations on how to handle and treat patients with initial COVID-19 symptoms.

## Data Availability Statement

All relevant data are reported in the manuscript and in the supplemental figures. All reasonable requests for other data details, restricted to non-identifying data owing to privacy concerns, can be requested by email from the corresponding author.

## Author Contributions

NP, SF, FDM, AL, FLL, RC, MR, GB, SP, and GR prepared the first draft of the manuscript. SF, FDM, FLL, RC, and MR collected the data. NP and GR finalized the manuscript based on comments and feedbacks from authors SF, FDM, AL, FLL, RC, MR, AG, AR, PR, CLV, GB, and SP. All authors gave final approval for the submission of the manuscript.

## Conflict of Interest

The authors declare that the research was conducted in the absence of any commercial or financial relationships that could be construed as a potential conflict of interest. The reviewer AG declared a shared affiliation, with no collaboration, with one of the authors, AL, to the handling editor at the time of the review.
