# Supplementary figures and images for: Bergamo and Covid-19: How the Dark Can Turn to Light
Source: Front Med (Lausanne). 2021 Feb 19;8:609440. doi: 10.3389/fmed.2021.609440 (PMC7933506; doi:10.3389/fmed.2021.609440)

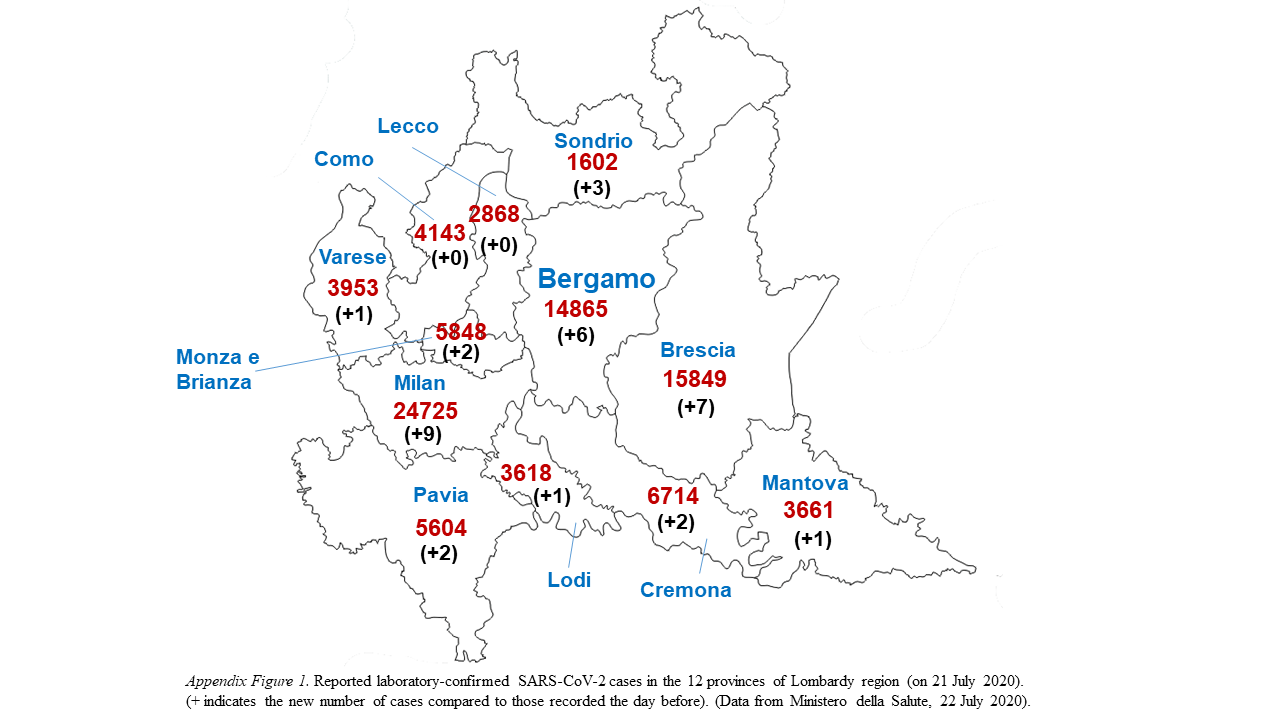

Supplement: Supplementary file 1 [file Image_1.tif]

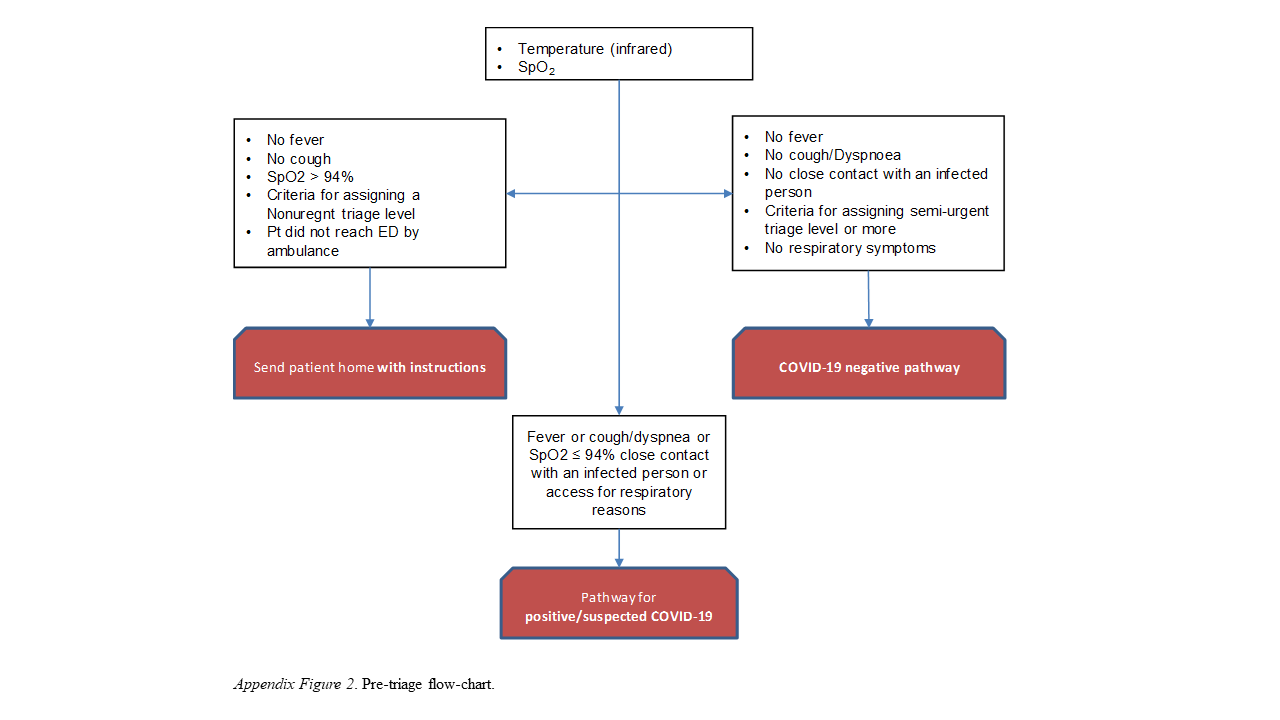

Supplement: Supplementary file 2 [file Image_2.tif]

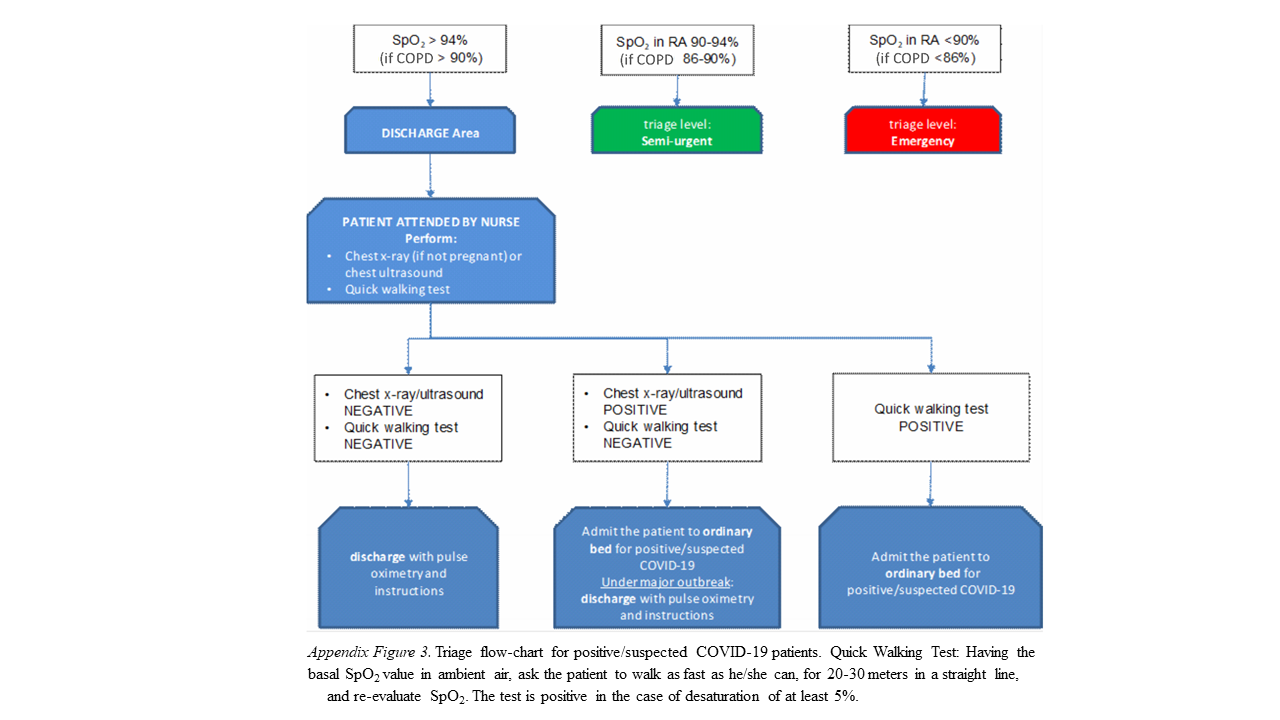

Supplement: Supplementary file 3 [file Image_3.tif]
