# Supplementary material for: Bergamo and Covid-19: How the Dark Can Turn to Light
Source: Front Med (Lausanne). 2021 Feb 19;8:609440. doi: 10.3389/fmed.2021.609440 (PMC7933506; doi:10.3389/fmed.2021.609440)
Supplement: Supplementary file 4 [file Data_Sheet_1.docx]

**Appendix 1.**

**COVID-19 Crisis Unit at the ASST-Papa Giovanni XXIII, Bergamo**

* Pezzoli Fabio          Medical Director

* Rizzi Marco          Head Infectious Diseases Unit

* Cosentini Roberto       Head Emergency Unit

* Fagiuoli Stefano         Head Department of Medicine

* Di Marco Fabiano       Head Respiratory Unit

* Lorini Luca Ferdinando     Head Department of Intensive Care Medicine and Emergency

* Cesa Simonetta         Head Department of Healthcare Professions

* Cacciabue Eleonora Head Healthcare Coordination

 * Bombardieri Giulia Medical Doctor Healthcare Coordination

* Stasi Beatrice         Chief Executive Officer

* Limonta Fabrizio        Health and Community Chief

* Fumagalli Monica        Chief Financial Officer

* Frattini Sabrina         Assistant Chief Executive Officer

* Spada Chiara          Nurse in Charge Medicine, Oncology and Hematology

* Tomasoni Laura        Nurse in Charge Surgical Departments

* Ferrari Maddalena       Nurse in Charge Operating Rooms

* Rota Lauretta          Nurse in Charge Emergency

* Ghilardi Patrizia        Nurse in Charge Gynecology

* Zanotti Anna          Nurse in Charge Bed Manager

* Casati Monica         Research, Education and Development Unit

* Capelli Cinzia         Bed Manager

* Daminelli Marinella       Nurse in Charge Pharmacy

* Daleffe Luigi          Nurse in Charge Risk Management

* Caldara Cristina        Nurse in Charge Health and Community

* Pagani Gabriele Medical Doctor Healthcare Coordination

* Scetti Silvia Medical Doctor Healthcare Coordination

* Canini Silvia Medical Doctor Healthcare Coordination

* Piccichè Antonio Medical Doctor Healthcare Coordination

* Cannistraro Valeria Medical Doctor Healthcare Coordination

* Colledan Michele        Director of the Transplant Department

* D'Antiga Lorenzo    Head Pediatric Unit

* Farina Claudio         Head Microbiology Unit

* Tramontini Mario        Head Internal Medicine 2 Unit

**Special persons involved in COVID-19 at the ASST-Papa Giovanni XXIII, Bergamo**

  * Gianatti Andrea Head Department of Laboratory Medicine
 * Sonzogni Aurelio Pathology, Department of Laboratory Medicine
 * Senni Michele Head Department of Cardiovascular diseases

  * Marchesi Mariano Intensive Care Unit 3, Department of Intensive Care

Medicine and Emergency

 * Ruggenenti Piero Director, Nephrology Unit, Department of Medicine

* Rambaldi Alessandro Director, Hematology Unit, Department of Oncology and

Oncohematology

* Sironi Sandro Head Department of Radiology

* Brembilla Paolo Department of Radiology
